# Blind Deconvolution Based on Correlation Spectral Negentropy for Bearing Fault

**DOI:** 10.3390/e25030543

**Published:** 2023-03-21

**Authors:** Tian Tian, Gui-Ji Tang, Yin-Chu Tian, Xiao-Long Wang

**Affiliations:** School of Energy, Power and Mechanical Engineering, North China Electric Power University, Baoding 071000, China

**Keywords:** blind deconvolution, correlation spectral negentropy, particle swarm optimization algorithm, harmonic interference, random pulse, feature extraction

## Abstract

Blind deconvolution is a method that can effectively improve the fault characteristics of rolling bearings. However, the existing blind deconvolution methods have shortcomings in practical applications. The minimum entropy deconvolution (MED) and the optimal minimum entropy deconvolution adjusted (OMEDA) are susceptible to extreme values. Furthermore, maximum correlated kurtosis deconvolution (MCKD) and multipoint optimal minimum entropy deconvolution adjusted (MOMEDA) are required prior knowledge of faults. On the basis of the periodicity and impact of bearing fault signals, a new deconvolution algorithm, namely one based on maximum correlation spectral negentropy (CSNE), which adopts the particle swarm optimization (PSO) algorithm to solve the filter coefficients, is proposed in this paper. Verified by the simulated vibration model signal and the experimental simulation signal, the PSO–CSNE algorithm proposed in this paper overcomes the influence of harmonic signals and random pulse signals more effectively than other blind deconvolution algorithms when prior knowledge of the fault is unknown.

## 1. Introduction

The rolling bearing is one of the key components of rotating machinery. Accidents of machine crashes and deaths due to bearing failures occur from time to time, so it is of great significance for the state detection and fault diagnosis of rolling bearings [1,2,3]. However, due to the complexity of the working conditions, the measured vibration signal is non–stationary, and the impact component of the early fault bearing vibration signal reflecting the fault characteristics is relatively weak. The blind deconvolution methods can extract the periodic pulse pattern of the fault bearing, so they are widely used in bearing fault diagnosis.

The minimum entropy deconvolution (MED) method allows blind deconvolution of vibration signals, thus eliminating the effect of the transfer path and highlighting the shock signal in the signal [4]. MED is widely used in fault diagnosis. H. Endo [5] used MED to enhance the filtering technique on the basis of the autoregressive model to improve its fault detection results. N. Sawalhi [6] combined MED and spectral kurtosis to improve the results of envelope analysis in fault diagnosis. Although MED can reduce the shock pulse signal, kurtosis is very sensitive to random pulses and is susceptible to interference from random pulses. The maximum correlation kurtosis deconvolution (MCKD) finds the optimal filter coefficients to maximize the correlation kurtosis [7]. Compared with MED, MCKD can extract the periodic pulse components effectively. MCKD needs to set the fault period when used, and it cannot obtain the fault period adaptively. Miao et al. [8] calculated the autocorrelation of the envelope signal to estimate the iteration period. Zhang [9] adaptively selected key parameters in MCKD using the grasshopper optimization algorithm. To eliminate the limitations of the MCKD method, McDonald [10] proposed the optimal minimum entropy deconvolution adjusted (OMEDA) and multipoint optimal minimum entropy deconvolution adjusted (MOMEDA) methods. OMEDA is based on the D–Norm criterion, which can maximize the convolution of individual pulses in the signal without an iterative process, but OMEDA is susceptible to noise. MOMEDA is based on the multi–point D–Norm criterion, which introduces a target vector to achieve accurate extraction of continuous multi–point shock components in bearing fault signals. However, the deconvolution results of MOMEDA depend on the a priori knowledge of the fault period. Xiao [11] determined the fault period by using MKurt and calculated the filter size of MOMEDA by using the advance–retreat algorithm. Cheng [12,13] optimized MED, MCKD, OMEDA, and MOMEDA by solving the filter coefficients with a standard particle swarm optimization algorithm based on generalized spherical coordinate transformation. Buzzoni [14] introduced a novel blind deconvolution algorithm based on an indicator of cyclostationarity The indicator has a high periodicity and can effectively eliminate the effect of random pulses. However, one of the input parameters of this algorithm requires an exact cycle frequency. To solve this problem, Zhang [15] proposed an adaptive maximum second–order cyclostationarity blind deconvolution (ACYCBD) to estimate the cycle frequency using envelope harmonic product spectrum (EHPS).

In this paper, a novel blind deconvolution method is proposed to overcome the difficulties applied to rolling bearing fault diagnosis. Correlation spectral negentropy is not only not susceptible to harmonics and random pulses but also has periodicity, and its selection as a criterion does not require a priori knowledge of the fault period. In addition, the new blind deconvolution algorithm has the advantage of fewer input parameters, and the results are less affected by the input parameters.

The remainder of this paper is structured as follows. Section 2 presents the specific introduction of the blind deconvolution based on maximum correlation spectral negentropy. Section 3 describes the details of the overall framework of the presented methodology. In Section 4 and Section 5, simulation model signal and experimental data are analyzed to investigate the effectiveness of the proposed methodology. Finally, Section 6 draws the conclusions.

## 2. The Blind Deconvolution Method

### 2.1. The Method of Blind Deconvolution

The core of the blind deconvolution problem is to design an inverse filter **f** to extract the periodic pulse characteristics **d_o_** from the fault signal **x**. That is, to deconvolve **x**:

(1) $$d=x*f=(d_{o}*h)*f\approx d_{o},$$

where **h** is the unknown transmission path, **f** is the finite impulse response (FIR) filter, **d** is the estimated fault feature information, and ∗ is the convolution operation.

The bearing fault signal **x** can be regarded as composed of multiple components:

(2) $$x=h_{d}*d_{o}+h_{u}*u+h_{n}*n,$$

(3) $$x=\left[\begin{array}{l} x_{1}\\ x_{2}\\ \vdots \\ x_{N} \end{array}\right],d_{o}=\left[\begin{array}{l} d_{1}\\ d_{2}\\ \vdots \\ d_{N} \end{array}\right],u=\left[\begin{array}{l} u_{1}\\ u_{2}\\ \vdots \\ u_{N} \end{array}\right],$$

where **x** denotes the bearing fault signal, **d_o_** means the periodic fault component, **u** is the unknown other component, **n** is the Gaussian white noise component, and **h_d_**, **h_u_**, and **h_n_** are the corresponding vibration transmission paths. 

Substituting Formula (2) into Formula (1), the theoretical formula for blind deconvolution in bearing fault diagnosis can be obtained:

(4) $$d=(h_{d}*d_{o}+h_{u}*u+h_{n}*n)*f\approx d_{o},$$

(5) $$f=\left[\begin{array}{l} f_{1}\\ f_{2}\\ \vdots \\ f_{L} \end{array}\right],$$

### 2.2. The Proposed Blind Deconvolution Method

#### 2.2.1. A New Criterion of Blind Deconvolution

Periodic pulses in the vibration signal are caused by bearing failure. A correlation exists between the pulse produced by the preceding hit and the pulse produced by the following impact. The signal’s autocorrelation may draw attention to its periodic character. In contrast, it is challenging to identify comparable elements in the random pulse and noise components, and there is little autocorrelation. The more visible the fault information is, the higher the negentropy value is. Therefore, in this paper, the correlation spectral negentropy [16] was chosen as the blind deconvolution indicator.

If the signal is *x*(*t*) and x(f;ω,Δω) denotes the complex envelope of the signal in the frequency domain of [ω−Δω/2;ω+Δω/2], then the energy in this frequency domain can be represented by the squared envelope as

(6) $$\varepsilon_{x}\left(t;\omega ,\Delta \omega \right)={\left|x(t;\omega ,\Delta \omega )\right|}^{2},$$

The unbiased autocorrelation, calculated on the basis of the squared envelope, can be defined as

(7) $${\hat{R}}_{xx}(\tau ;\omega ,\Delta \omega )=\frac{1}{N-q}\sum_{j=1}^{N-q}{\left|x(t_{j};\omega ,\Delta \omega )\right|}^{2}{\left|x(t_{j}+\tau ;\omega ,\Delta \omega )\right|}^{2},$$

where $\tau =q/f_{s}$ means the delay factor, and *q* = 0, 1, 2, 3…*N* − 1.

The instantaneous energy obtained by the squared envelope can be described as

(8) $$\varepsilon_{R}(f;\omega ,\Delta \omega )=\left|{\hat{R}}_{XX}{}^{\prime }(f;\omega ,\Delta \omega )\right|,$$

where $\left|{\hat{R}}_{XX}{}^{\prime }(f;\omega ,\Delta \omega )\right|$ denotes the complex envelope in the frequency domain.

Correlation spectral negentropy (CSNE) that is analyzed in this paper can be expressed as

(9) $$\Delta I_{\varepsilon }(\omega ;\Delta \omega )=-H_{\omega }(\omega ;\Delta \omega )=-\left\{-\langle \frac{\varepsilon_{R}{\left(f;\omega ,\Delta \omega \right)}^{2}}{\langle \varepsilon_{R}{\left(f;\omega ,\Delta \omega \right)}^{2}\rangle }\ln\frac{\varepsilon_{R}{\left(f;\omega ,\Delta \omega \right)}^{2}}{\langle \varepsilon_{R}{\left(f;\omega ,\Delta \omega \right)}^{2}\rangle }\rangle \right\},$$

#### 2.2.2. Maximization Criterion Based on Correlation Spectral Negentropy

By improving the finite impulse response (FIR) filter, the blind deconvolution approach based on maximum CSNE seeks to remove the impact of the transmission path. It is assumed that a FIR filter is employed to maximize the CSNE, which will raise the fault pulse with a high CSNE in the signal while lowering noise and other associated components with a low CSNE. The filter **f** is the result of the following maximization problem:

(10) $$\max\mathrm{CSNE}=\max\Delta I_{\varepsilon }(\omega ;\Delta \omega ),$$

Inspired by [12], particle swarm optimization (PSO) algorithm was utilized to optimize the solution of the inverse filter. On the basis of the observation of the activity behavior of bird clusters, the PSO algorithm uses the sharing of information by individuals in the group to move the whole group, evolving from disorder to order in the problem–solving space, to obtain the optimal solution. As a global optimization algorithm, PSO algorithm is particularly suitable for solving optimization problems with high–dimensional complex structures. The inverse filter with better performance is solved using PSO, and CSNE is used as the objective function for optimization.

Using the generalized spherical coordinate transformation, the filter $f={\left[f_{1},f_{2},\cdots ,f_{L}\right]}^{T}$ can be expressed as

(11) $$\left\{ \begin{array}{l} f_{1}=\cos(\theta_{1})\\ f_{2}=\sin(\theta_{1})\cos(\theta_{2})\\ \vdots \\ f_{L-1}=\sin(\theta_{1})\cdots \sin(\theta_{L-2})\cos(\theta_{L-1})\\ f_{L}=\sin(\theta_{1})\cdots \sin(\theta_{L-2})\sin(\theta_{L-1}) \end{array}\right.-\pi \leq \theta_{s}\leq \pi ,s=1,2,\cdots ,L-1,$$

where *L* is the length of the filter, and *θs* means the angel parameter in the range of [–π, π].

The search space for deconvolution is

(12) $$\Omega^{L-1}=[-\pi ,\pi ]\times [-\pi ,\pi ]\times \cdots [-\pi ,\pi ]\subset R^{L-1},$$

Then, the deconvolution problem becomes the following optimization problem:

(13) $$\Theta_{opt}\left\{\mathrm{CSNE}[f(\Theta_{opt})]\right\}\geq \mathrm{CSNE}[f(\Theta )],\forall \Theta \in \Omega^{L-1},$$

where $\Theta =[\theta_{1},\theta_{2},\cdots ,\theta_{L-1}]$ denotes the angle vector, and CSNE(f) represents the CSNE of the filtered signal.

#### 2.2.3. The Optimization of the Filter Length *L*

The kurtosis is particularly sensitive to impact. The larger the kurtosis value is, the more the impact the component accounts for. If the fault frequency in the envelope spectrum is prominent, the kurtosis value of the envelope spectrum is larger. Therefore, the kurtosis of envelope spectrum (ESK) [17] is used to measure the deconvolution effect of the original signal by PSO–CSNE. ESK can be described as

(14) $$\mathrm{ESK}=\frac{\sum_{n=1}^{N}{\mathrm{ES}}_{n}^{4}}{{\left(\sum_{n=1}^{N}{\mathrm{ES}}_{n}^{2}\right)}^{2}},$$

where ES means the envelope spectrum of the original signal, and *N* denotes the length of the original signal.

Set the optimization range of the filter length *L* to [10, 200], set the step size to 10, calculate the ESK of the signal after deconvolution with different *L* values, and take the corresponding one when the ESK is the largest. The filter length is the optimal value.

## 3. The Proposed Fault Diagnosis Framework

**Figure 1:**
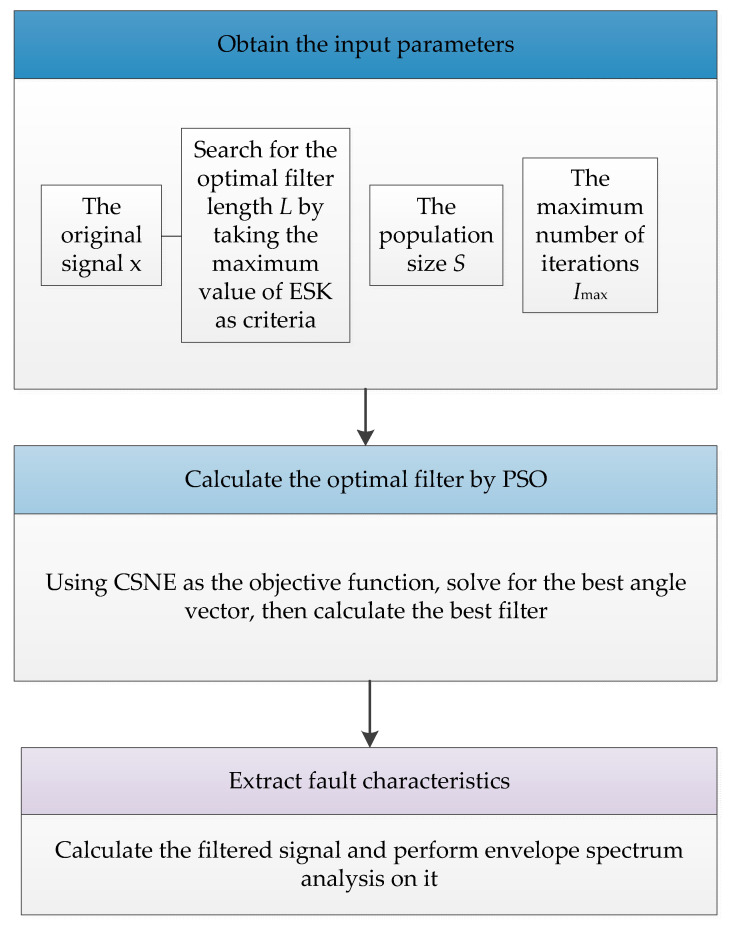
The block diagram of the proposed fault diagnosis framework.

In order to solve the problem of bearing fault feature extraction affected by harmonics and random pulses, this paper proposes a blind deconvolution algorithm based on the maximum correlation spectral negentropy. Its flowchart is depicted in Figure 1, and the specific description of the corresponding steps is given as follows:

Step 1: Input the fault signal **x**, search for the filter length *L*, and determine the optimal value, taking the maximum value of the envelope spectrum kurtosis as the evaluation standard.

Step 2: Load the fault signal **x**, the optimal filter length *L*, the population size *S*, and the maximum number of iterations *I*_max_.

Step 3: Use the PSO method to solve the optimization problem of Equation (13) and obtain the best angle vector $\Theta_{\mathrm{opt}}$.

Step 4: Calculate the optimal filter **f**_opt_ according to Formula (11) and obtain the filtered signal **d**.

Step 5: Perform envelope spectrum analysis on the filtered signal **d.**

## 4. Simulation Signal Analysis

In order to verify the effectiveness of the method, simulated bearing fault signals were used. In addition to the periodic pulse signal *o*(*t*), the bearing simulation signal may also include the harmonic signal *h*(*t*), the random pulse signal *r*(*t*), and the Gaussian white noise signal *n*(*t*). The sampling frequency is 9000, and the number of sampling points is 4096.

The periodic pulse signal *o*(*t*) produced by outer race defect is described as follows [18], and its parameters are listed in Table 1:

(15) $$o(t)=\left[\sum_{i=0}^{M-1}Dh(t-iT_{o})\right]*[A_{1}e^{-\xi 2\pi f_{n}t}\cdot \cos(2\pi f_{d}t)],$$

where *M* denotes the number of impulses, *D* means single pulse intensity, *A*_1_ is amplitude, *ξ* is system damping ratio, *f_n_* and *f_d_* mean the natural frequency and the resonance frequency, respectively, and *T*_0_ means the fault period.

The harmonic signal *h*(*t*) can be formulated as follows [19], and its parameters are shown in Table 2:

(16) $$h(t)=A_{2}\sin(2\pi f_{1}t)+A_{3}[0.6+0.5\sin(2\pi f_{2}t)]+A_{4}\sin(2\pi f_{3}t-\pi /3),$$

The random pulse signal *r*(*t*) is formulated as follows [20], where *M*_1_ means the number of random impulses and is set to 1, and *P_j_* denotes the amplitude of *j*–th random impulse and *P*_1_ is set to 8. The parameters of system damping ratio *ξ*_1_, the natural frequency *f*_n1_, and the resonance frequency *f*_4_ are listed in Table 3.

(17) $$r(t)=\sum_{j=1}^{M_{1}}P_{j}e^{-\xi_{1}2\pi f_{n1}t}\sin(2\pi f_{4}t),$$

In order to prove the superiority of the deconvolution index selected in this paper, four indicator values of the periodic pulse signal, the harmonic signal, and the random pulse signal are compared. It can be seen from Table 4 that the kurtosis value and the Gini coefficient of the random pulse signal are larger than those of the periodic pulse, that is, the kurtosis and the Gini coefficient are easily affected by the random pulse. It can be observed that CSNE is not affected by either the harmonic signal or random pulse signal. It can be concluded that the CSNE performs better than the other two indicators in the case of unknown failure period.

### 4.1. The Vibration Model with Harmonic Interference

**Figure 2:**
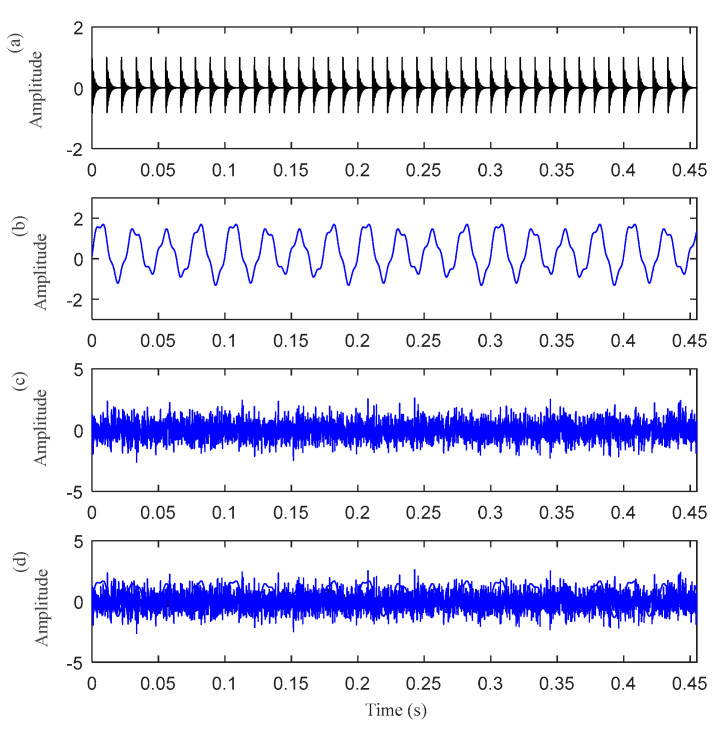
The time−–domain waveform of the simulated signal: (**a**) the periodic pulse signal *o*(*t*); (**b**) the harmonic signal *h*(*t*); (**c**) the Gaussian white noise signal *n*(*t*) (SNR = 3 dB); and (**d**) the simulated bearing outer ring fault signal *x*(*t*).

**Figure 3:**
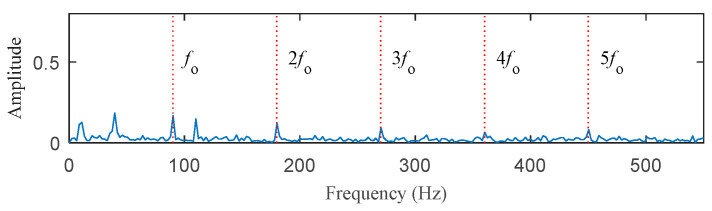
The envelope spectrum of the simulated signal *x*(*t*).

The first case is to analyze the simulation bearing with discrete harmonic interference. The time–domain waveform of the simulated signal is shown in Figure 2. Figure 2a–c are the time–domain waveform diagrams of the periodic pulse signal *o*(*t*), the harmonic signal *h*(*t*), and the Gaussian white noise signal *n*(*t*). Figure 2d shows the simulated bearing outer ring fault signal synthesized by these three signals. It can be seen from Figure 2d that periodic pulses are submerged by the other two signals. Figure 3 is the envelope spectrum of the fault signal *x*(*t*). Although there are peaks at *f*_o_ and its harmonic frequencies, their amplitude is low (The five red dotted lines refer to *f*_o_, 2*f*_o_, 3*f*_o_, 4*f*_o_, and 5*f*_o_, respectively).

**Figure 4:**
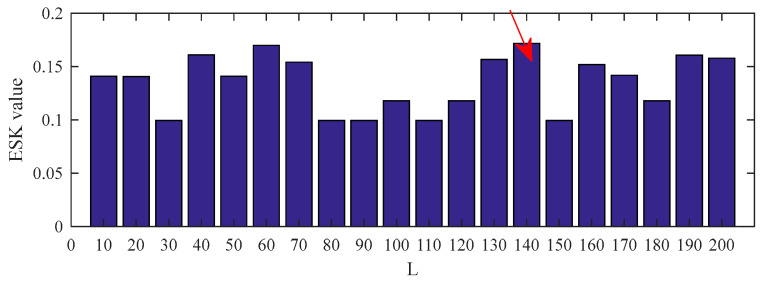
ESK value of the PSO–CSNE filtered signal under different filter lengths. (The red arrow in the figure shows the filter length with the maximum ESK value.)

**Figure 5:**
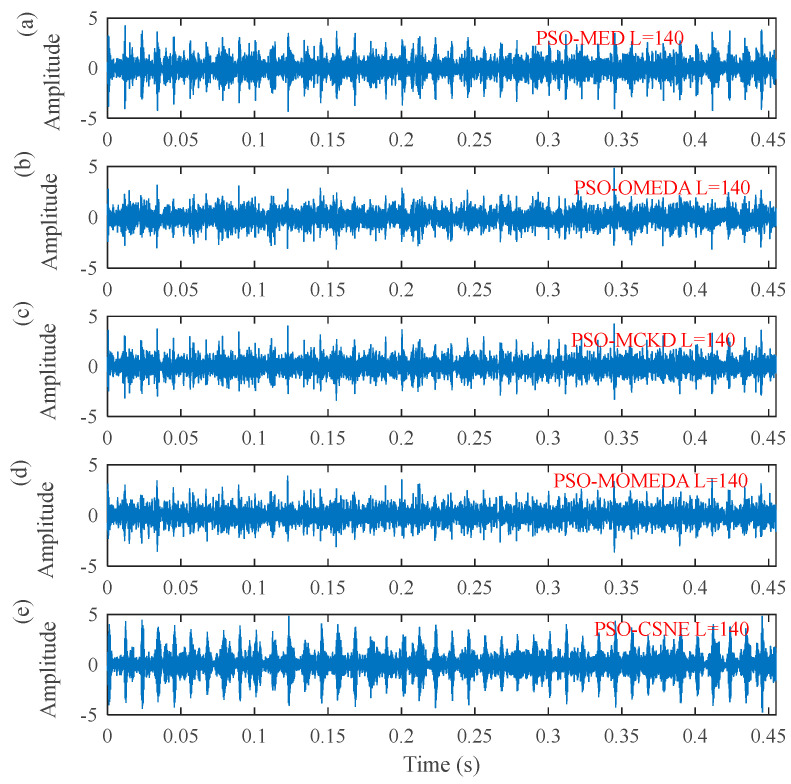
The time–domain waveforms after five deconvolution methods: (**a**) PSO–MED; (**b**) PSO–OMEDA; (**c**) PSO–MCKD; (**d**) PSO–MOMEDA; and (**e**) PSO–CSNE.

**Figure 6:**
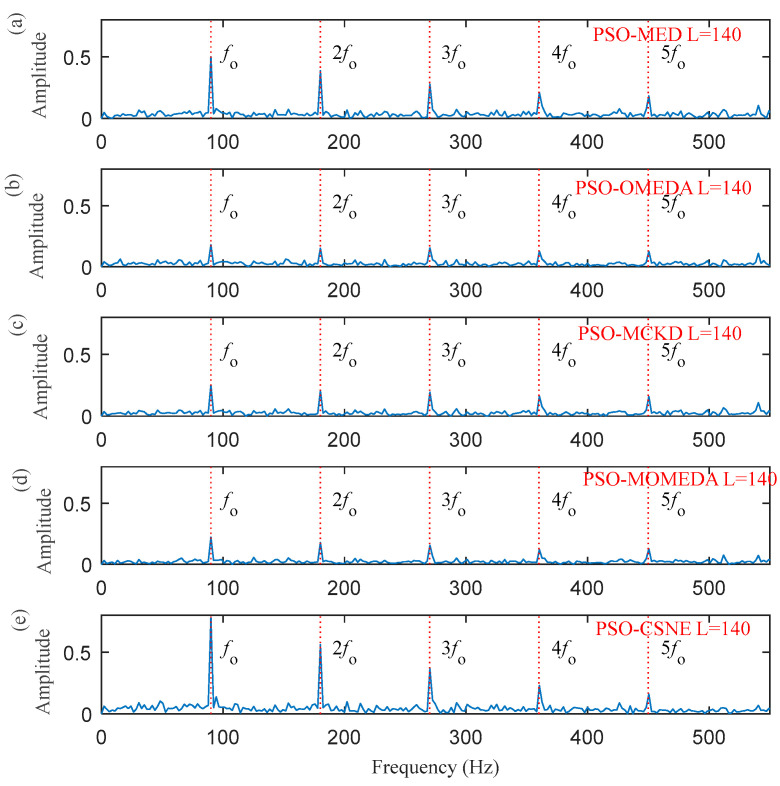
The envelope spectrum of the filtered signals using five deconvolution methods: (**a**) PSO–MED; (**b**) PSO–OMEDA; (**c**) PSO–MCKD; (**d**) PSO–MOMEDA; and (**e**) PSO–CSNE.

Figure 4 shows the ESK value of the PSO–CSNE filtered signal under different filter lengths. According to Figure 4, when the filter length is 140, the ESK value is the largest. Therefore, the filter length is selected to 140. The results were compared with the results of the PSO–MED, PSO–OMEDA, PSO–MCKD, and PSO–MOMEDA methods to highlight the advantages of PSO–CSNE. The filtered signals of five deconvolution methods are shown in Figure 5. It can be observed that the periodic pulses in PSO–OMEDA are almost submerged by noise, and the periodic pulses in PSO–MCKD and PSO–MOMEDA are also not obvious enough. The results show that PSO–MED and PSO–CSNE are not affected by the harmonic component and noise. The envelope spectrums of filtered signal using the five deconvolution methods are described in Figure 6. As can be observed from Figure 6, although the outer ring failure frequency *f*_o_ and its harmonics can be observed in the envelope spectrum of all the five algorithms, the amplitudes of PSO–MED and PSO–CSNE are larger than those of the other three deconvolution algorithms.

### 4.2. The Vibration Model with Both Harmonic Interference and Random Impulse Interference

**Figure 7:**
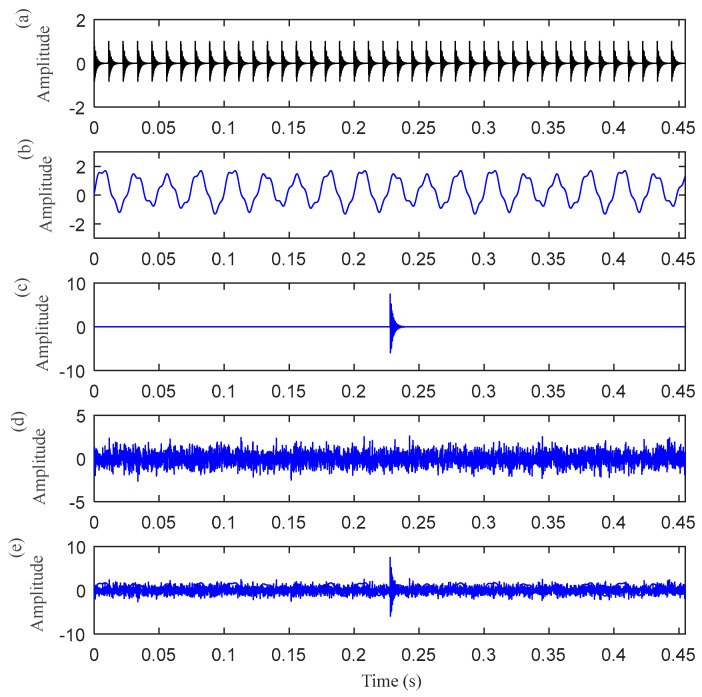
The time–domain waveform of the simulated signal: (**a**) the periodic pulse signal *o*(*t*); (**b**) the harmonic signal *h*(*t*); (**c**) the random pulse signal *r*(*t*); (**d**) the Gaussian white noise signal *n*(*t*) (SNR = 3 dB); and (**e**) the simulated bearing outer ring fault signal *x*(*t*).

**Figure 8:**
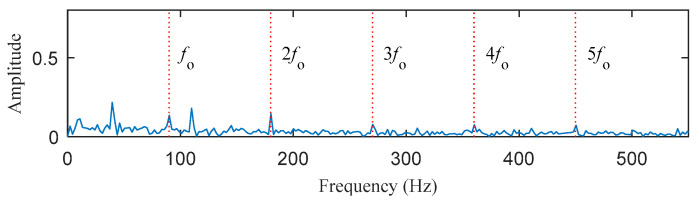
The envelope spectrum of the simulated signal *x*(*t*).

In this section, a case of simulated signal with both harmonic interference and random pulse interference was employed to evaluate the performance of the proposed method. As shown in Figure 7, the mixed signal *x*(*t*) consists of the periodic impulse signal *o*(*t*) produced by the outer race, harmonic signal *h*(*t*), random impulse signal *r*(*t*), and Gaussian white noise signal *n*(*t*). It can be seen from Figure 7d that the periodic impulse is masked by harmonics interference, random pulse, and noise. Affected by the interference component, the outer race fault characteristic frequency in the envelope spectrum is not obvious, which is not enough to diagnose the fault, as shown in Figure 8.

**Figure 9:**
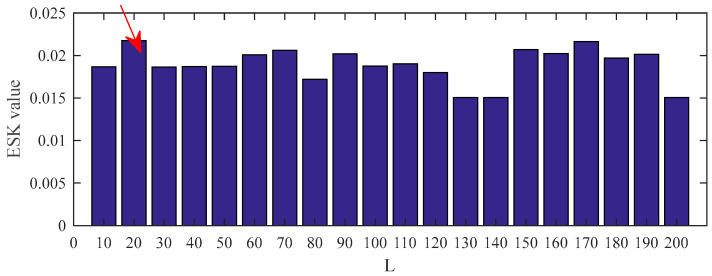
ESK value of the PSO–CSNE filtered signal under different filter lengths. (The red arrow in the figure shows the filter length with the maximum ESK value.)

**Figure 10:**
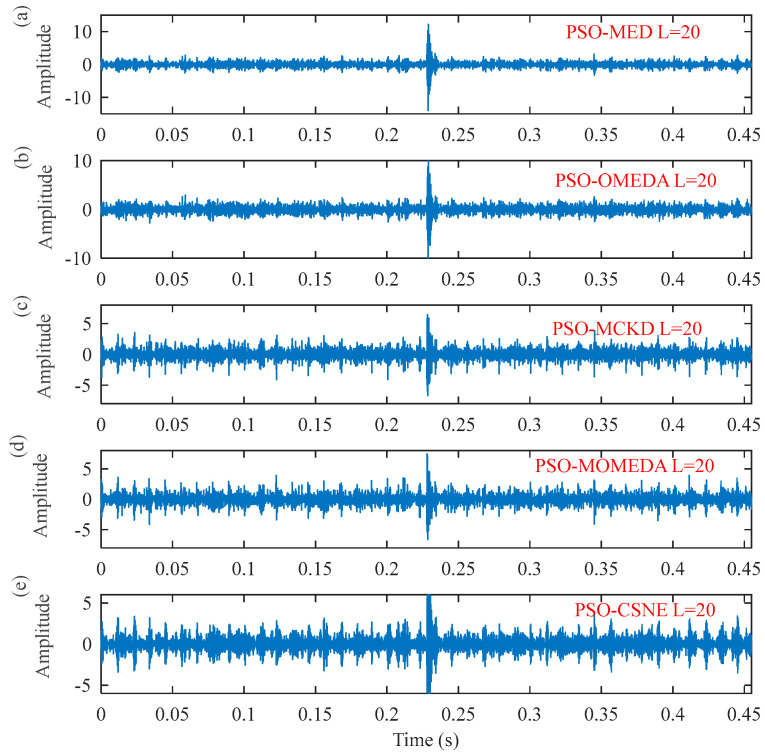
The time–domain waveforms after five deconvolution methods: (**a**) PSO–MED; (**b**) PSO–OMEDA; (**c**) PSO–MCKD; (**d**) PSO–MOMEDA; and (**e**) PSO–CSNE.

**Figure 11:**
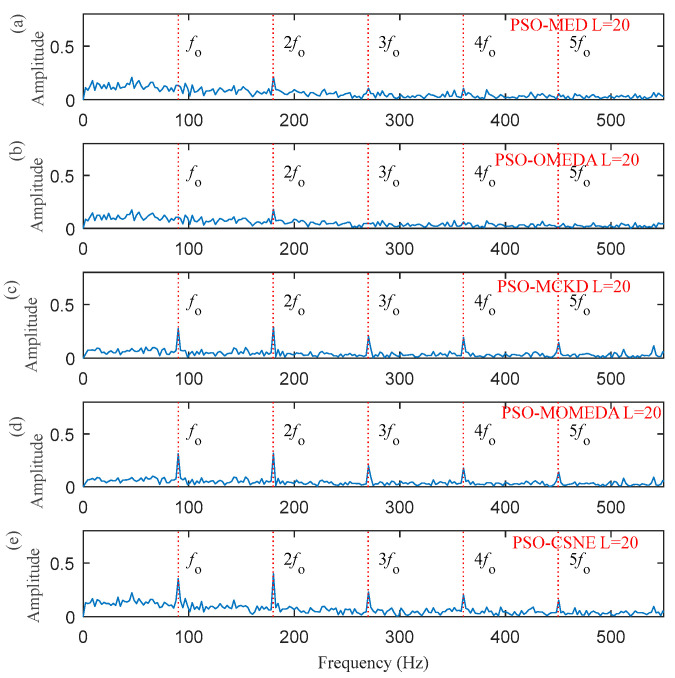
The envelope spectrum of the filtered signals using five deconvolution methods: (**a**) PSO–MED; (**b**) PSO–OMEDA; (**c**) PSO–MCKD; (**d**) PSO–MOMEDA; and (**e**) PSO–CSNE.

The result in Figure 9 demonstrates that the optimal filter length is 20. Thus, setting the length of the filter to 20, the results of using the five deconvolution methods are illustrated in Figure 10 and Figure 11, respectively. Figure 10 plots the time–domain waveform of the filtered signals. The amplitudes of random impulse in filtered signals using PSO–MED and PSO–OMEDA are larger than the other methods. Furthermore, the result of PSO–CSNE has the lowest amplitude of random impulse and a more obvious periodicity. The envelope spectrums obtained using the PSO–MED and PSO–OMEDA have a poor performance, such that there is only 2*f*_o_ in the envelope spectrums; PSO–CSNE does not require prior knowledge of fault frequency but has more obvious fault frequencies and harmonics than PSO–MCKD and PSO–MOMEDA, which do require prior knowledge, as shown in Figure 11. 

## 5. Experiment Simulation Signal Analysis

The experimental data come from the Bearing Data Center of the University of Case Western Reserve [21]. The test bench includes a three–phase induction motor on the left, a torque sensor and coupling in the middle, and a dynamometer on the right. Acceleration sensors are installed on the motor drive end, fan end, and support base. The test bench can test two types of bearings, namely the SKF6205 bearing installed on the drive end of the motor and the SKF6203 bearing installed on the fan end. Electric spark technology is used to simulate the pitting phenomenon in engineering practice, process faults of different sizes in the inner rings, outer rings, and rollers of the rolling bearing.

### 5.1. Inner Race Fault at a Rotation Speed of 1730 rpm

**Figure 12:**
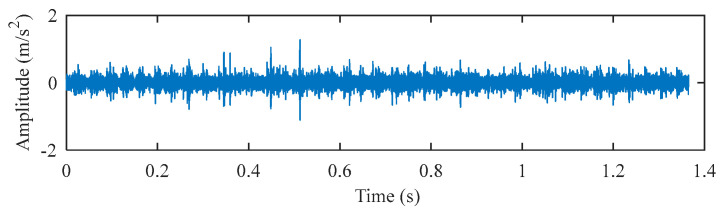
The time–domain waveform of the original signal.

**Figure 13:**
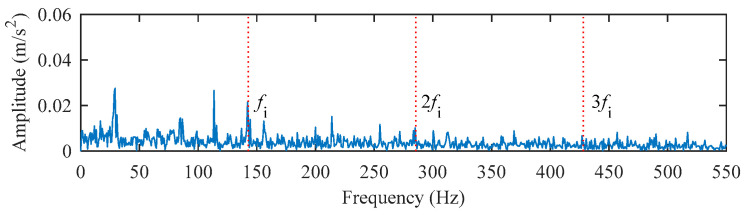
The envelope spectrum of the original signal.

The data of the inner ring fault bearing with a fault size of 0.014 inches at the fan end was selected for analysis. The motor speed is 1730 rpm, the sampling frequency is 12 kHz, and the inner ring fault characteristic frequency is 142.73 Hz. The middle segment containing 16384 continuous points with random pulses was selected. The time–domain waveform and envelope spectrum of the original signal before processing are displayed in Figure 12 and Figure 13. It can be seen that the original signal contains obvious random pulse components, the background noise is large, the impact characteristics are not obvious, and it is difficult to diagnose the fault from the envelope spectrum.

**Figure 14:**
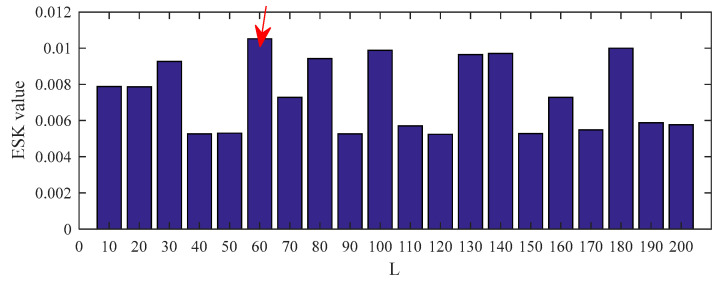
ESK value of the PSO–CSNE filtered signal under different filter lengths. (The red arrow in the figure shows the filter length with the maximum ESK value.)

**Figure 15:**
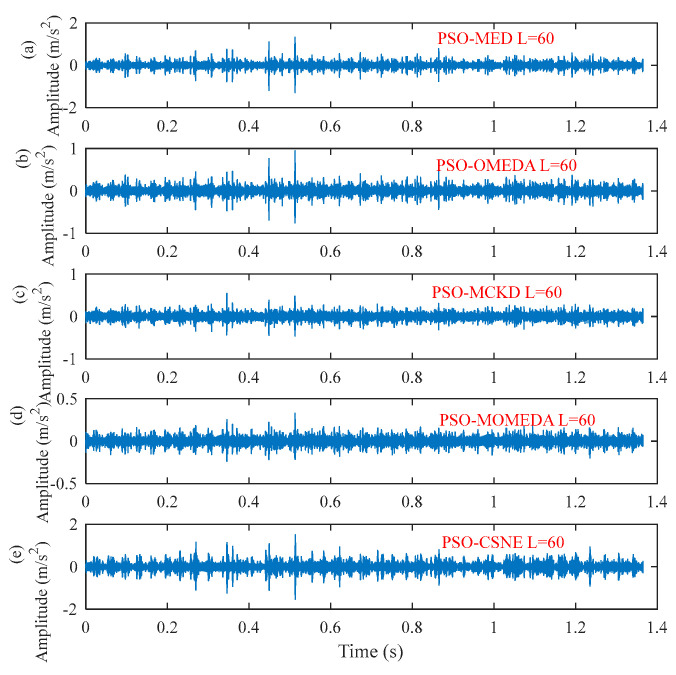
The time–domain waveforms after five deconvolution methods: (**a**) PSO–MED; (**b**) PSO–OMEDA; (**c**) PSO–MCKD; (**d**) PSO–MOMEDA; and (**e**) PSO–CSNE.

**Figure 16:**
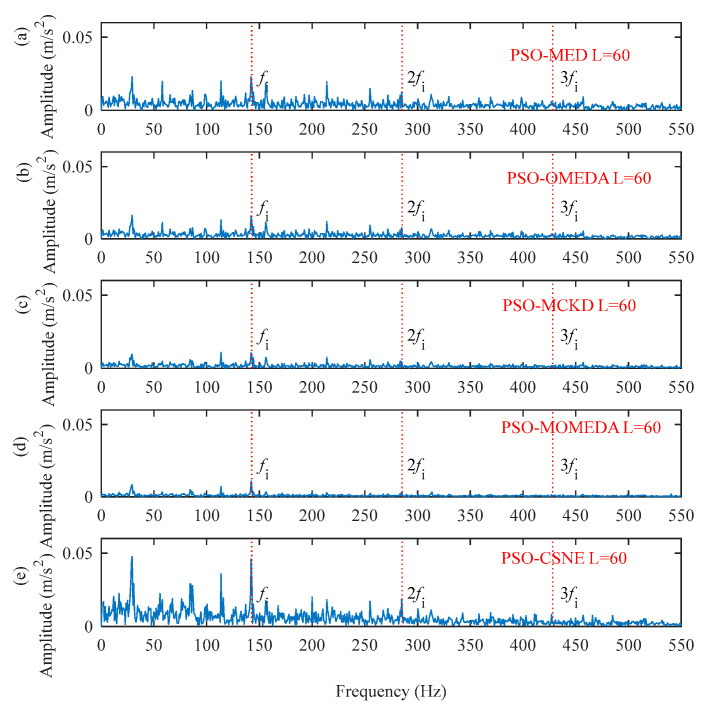
The envelope spectrum of the filtered signals using five deconvolution methods: (**a**) PSO–MED; (**b**) PSO–OMEDA; (**c**) PSO–MCKD; (**d**) PSO–MOMEDA; and (**e**) PSO–CSNE.

The filter length can be obtained from Figure 14. The deconvolution results of PSO–MED, PSO–OMEDA, PSO–MCKD, and PSO–MOMEDA are compared with that of the proposed method to prove its advantage. As can be seen from Figure 15, PSO–MED, PSO–OMEDA, PSO–MCKD, and PSO–MOMEDA have poor deconvolution performance because there are still obvious random pulses in the filtered signal. Although there is a random pulse in the filtered signal of PSO–CSNE, the periodic pulse can be observed. The envelope spectrum of the filtered signals using five deconvolution methods are shown in Figure 16. The peak at *f*_i_ can be observed in all envelope spectrums, but the amplitude of Figure 16e is larger than the others. In addition, there is a peak at 2*f*_i_ only in Figure 16a,e. Furthermore, the envelope spectrum of PSO–MED has peaks at *f*_i_ and 2*f*_i_, but there are too many interference components at surrounding frequencies. In summary, when there are random pulses in the signal, the deconvolution performance of PSO–CSNE performs better than the other four deconvolution algorithms (The five red dotted lines refer to *f*_i_, 2*f*_i,_ and 3*f*_i_, respectively). 

### 5.2. Roller FAULT at a Rotation Speed of 1772 rpm

**Figure 17:**
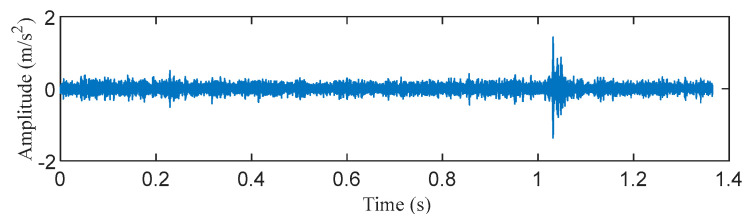
The time–domain waveform of the original signal.

**Figure 18:**
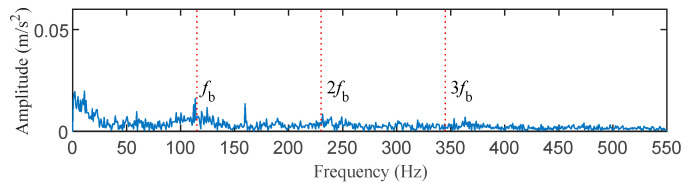
The envelope spectrum of the original signal.

The data of the roller fault bearing with a fault size of 0.014 inches at the fan end was selected for analysis to illustrate the superiority of the proposed method. The motor speed is 1772 rpm, the sampling frequency is 12 kHz, and the roller fault characteristic frequency is 117.84 Hz. The middle segment containing 16384 continuous points with random pulses was selected. The time–domain waveform and envelope spectrum of the original signal before processing are displayed in Figure 17 and Figure 18. It can be observed that the original signal contains an obvious random pulse, the background noise is heavy, and the impact characteristics are not obvious to diagnosis from the envelope spectrum.

**Figure 19:**
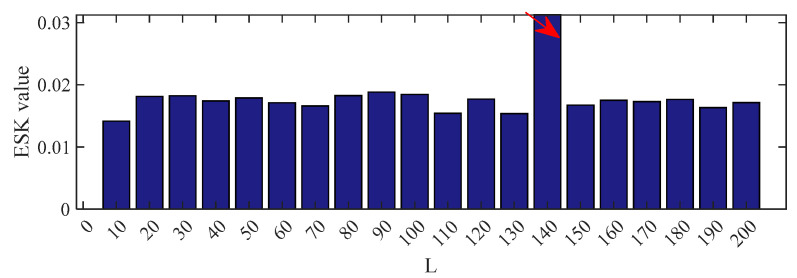
ESK value of the PSO–CSNE filtered signal under different filter lengths. (The red arrow in the figure shows the filter length with the maximum ESK value.)

**Figure 20:**
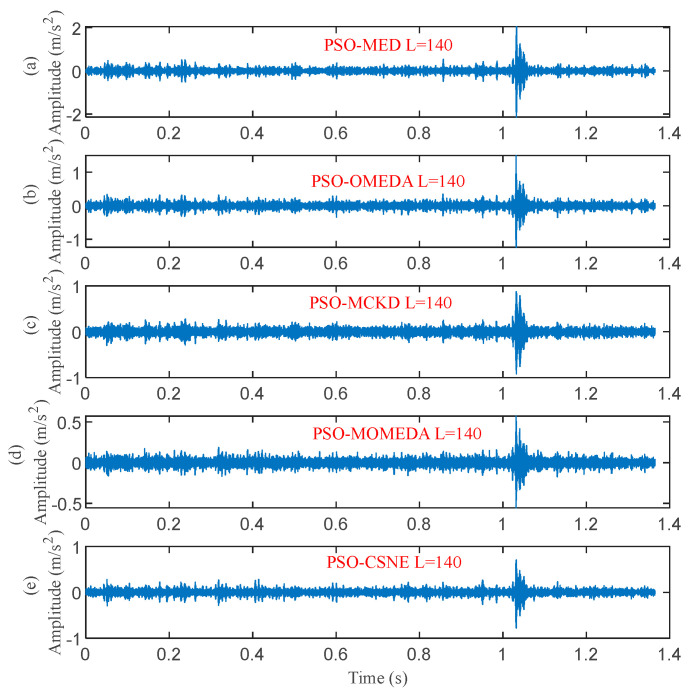
The time domain waveforms after five deconvolution methods: (**a**) PSO–MED; (**b**) PSO–OMEDA; (**c**) PSO–MCKD; (**d**) PSO–MOMEDA; and (**e**) PSO–CSNE.

**Figure 21:**
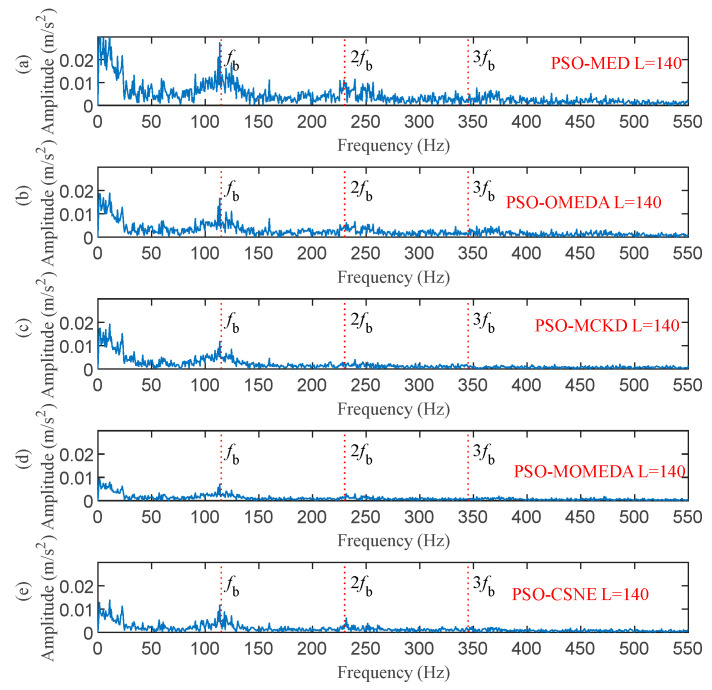
The envelope spectrum of the filtered signals using five deconvolution methods: (**a**) PSO–MED; (**b**) PSO–OMEDA; (**c**) PSO–MCKD; (**d**) PSO–MOMEDA; and (**e**) PSO–CSNE.

The filter length was set to 140 according to Figure 19. Figure 20 and Figure 21 show the outcomes of these five methods as a plot. The random pulse in Figure 20e is attenuated, despite the fact that it is still visible in the time domain plots of all five filtered signals. It is clear that the noise in Figure 21a,b interferes with the ability to distinguish between the fault characteristic frequencies and their multiples. In Figure 21c,d, only the fault characteristic frequency can be identified; the multiplier frequency cannot. Not only is roller fault frequency *f*_b_ identified in Figure 21e, but also is 2*f_b_*. (The five red dotted lines refer to *f*_b_, 2*f*_b,_ and 3*f*_b_, respectively). It demonstrates that PSO–CSNE outperforms the other four approaches in terms of deconvolution results.

## 6. Conclusions

A blind deconvolution method based on correlation spectral negentropy was proposed to address the shortcomings of existing blind deconvolution methods that are vulnerable to random pulses and require a priori knowledge. The envelope spectral kurtosis is used as an indicator to select the optimal filter length for the blind deconvolution method proposed in this paper. By comparing the envelope spectrum of the deconvoluted signals, it is demonstrated that the method in this paper can avoid the interference of harmonics and random pulses better than other blind deconvolution algorithms without the need for a priori knowledge. In this paper, only the effect of blind deconvolution for extracting fault features at smooth speed is discussed, and further improvement of blind deconvolution at variable speed will be discussed next.

## Figures and Tables

**Table 1:** Parameters of the period pulse signal *o*(*t*).

Parameter	*M*	*D*	*A*_1_	*ξ*	*f_n_* (Hz)	*f_d_* (Hz)	*T*_0_ (s)
Value	41	1	1	0.05	2000	1998	1/90

**Table 2:** Parameters of the discrete harmonic signal *d*(*t*).

Parameter	*A*_2_	*A*_3_	*A*_4_	*f*_1_ (Hz)	*f*_2_ (Hz)	*f*_3_ (Hz)
Value	1.2	0.5	0.2	40	30	150

**Table 3:** Parameters of the random impulse signal *r*(*t*).

Parameter	*P*_1_	*M*_1_	*ξ*_1_	*f_n_*_1_ (Hz)	*f*_4_ (Hz)
Value	8	1	0.03	2500	2499

**Table 4:** The comparison of three deconvolution indicators.

	Kurtosis	Gini	CSNE
*o*(*t*)	0.0028	0.7516	0.726
*h*(*t*)	0.0005	0.3476	0.0069
*r*(*t*)	0.0802	0.9921	0.5549

## Data Availability

Not applicable.
